# Signaling of Protein Kinases in Development and Disease

**DOI:** 10.3390/biomedicines14040788

**Published:** 2026-03-30

**Authors:** Zheng Fu

**Affiliations:** Department of Pharmacology, University of Virginia, Charlottesville, VA 22908, USA; zf6n@virginia.edu

Protein kinases are key mediators of signal transduction and central regulators of cellular functions. Their dysfunction underlies many human diseases. Given their critical role in pathogenesis, protein kinases are a major family of drug targets for pharmacological intervention. As of 2025, over 80 small-molecule protein kinase inhibitors had been approved by the FDA for the treatment of neoplasms and inflammatory diseases [1]. Artificial intelligence (AI)-based approaches and models have elevated the discovery and development of protein kinase inhibitors to a new level [2]. The use of deep generative models has not only shortened the turnaround time for the design and optimization of de novo small molecules but also enabled more accurate and robust predictions of multitargeted kinase inhibitors [3,4]. Despite these advances, therapeutic applications of protein kinase inhibitors have seen limited success due to our incomplete understanding of kinase signaling mechanisms and functions. In this context, the first edition of this Special Issue reported exciting new findings and perspectives on targeting protein kinase signaling pathways in cancer and chronic inflammation.

Acquisition of resistance to protein kinase inhibitors in cancer cells is still a major obstacle to achieving long-term success in cancer therapy. Targeted inhibition of a single kinase in signaling pathways always elicits resistance due to alternative and compensatory mechanisms. Multi-targeting interconnected kinase pathways have shown promise in overcoming resistance to achieve better therapeutic effects. In this Special Issue, Yusuff Olayiwola and Lauren Gollahon explored the application of phytochemicals—naturally occurring, bioactive compounds—as multi-tasking therapeutic agents to modulate key protein kinases and biological processes in breast cancer cells [5]. This in silico study showcased the power of computational bioinformatics in predicting the pharmacological profiles of drug candidates.

Reduction in *N*^6^-methyladenosine (m^6^A), the most common epitranscriptomic modification on mammalian mRNA, is a major non-genetic mechanism underlying the intrinsic and acquired resistance to tyrosine kinases inhibitors (TKIs) [6]. Aayush Rastogi and colleagues demonstrated that resistance to EGFR tyrosine kinase inhibitors in lung cancer cells can be ameliorated by downregulating the m^6^A demethylase FTO (fat mass- and obesity-associated) [7]. Furthermore, FTO overexpression can be a potential prognostic marker in NSCLC (non-small-cell lung cancer) patients. This study underscores the promise of targeting FTO to overcome the TKI resistance phenotype in lung cancer.

Many natural compounds with antioxidant and anti-inflammatory properties have shown great potential in the treatment of human diseases. Aline Müller and colleagues discovered that a natural oil compound, farnesol, modulates TNF-α/IL-1β-induced phosphorylation of PI3K and its downstream signaling proteins to suppress inflammatory gene expression in human renal epithelial cells [8]. This result implicates the strong potential of using farnesol to treat chronic kidney disease (CKD).

Cytokinesis, a highly ordered process in the last stage of cell division, is tightly controlled through phosphorylation by mitotic kinases, such as Aurora B, polo-like kinase 1, and cyclin-dependent kinase 1 [9]. Recently, the roles of other kinases (e.g., casein kinase 2, checkpoint kinase 2, and p21-activated kinase) in cytokinesis have also been identified [10]. Ursula Braun and Michael Leitges published compelling evidence that the depletion of protein kinase D3 (PKD3) leads to failed abscission and multinucleation, highlighting the requirement of a previously unrecognized PKCε-PKD3 pathway in the control of cytokinesis [11].

Finally, Michael Lemke and his colleagues presented an insightful perspective on the evolution, structure, regulation, and function of MAST (microtubule-associated serine/threonine) kinases by integrating bioinformatic analysis, predictive algorithms, and AlphaFold [12]. Their work produced valuable structural models for investigating the molecular mechanism and functional impact of human disease mutations in MAST kinases.

Many new developments have recently emerged as a result of significant advances in knowledge and technology. Phase separation of protein kinases presents new challenges for understanding kinase regulation and functions in large membrane-less biomolecular condensates [13,14]. Given the critical role of intrinsically disordered regions (IDRs) in protein phase separation [15], small-molecule inhibitors targeting the non-catalytic IDR of protein kinases are desirable, as they can disrupt specific functions, achieve higher selectivity, and reduce off-target toxicity. AI-assisted design enables the targeting of specific kinase isoforms and conformational states to reduce off-target effects, accelerating the development of more effective kinase inhibitors and activators [16]. AI analytics can also improve accuracy in mapping kinase substrates and cofactors, as well as in predicting the functional effects of human mutations. Recent advancements in mass spectrometry and biosensor imaging technology allow for better profiling of kinase signaling networks and real-time visualization of kinase activity in living single cells [17,18,19,20].

In conclusion, this Special Issue serves as an open access platform to showcase recent work toward a deeper understanding of protein kinases for therapeutic applications. I hope that the second edition will continue to advance our understanding of kinase signaling mechanisms and networks in the context of human diseases.

## Abbreviations

The following abbreviations are used in this manuscript:

FDA	Food and Drug Administration
AI	Artificial Intelligence
TKI	Tyrosine Kinase Inhibitor
EGFR	Epidermal Growth Factor Receptor
FTO	Fat Mass- and Obesity-Associated
NSCLC	Non-Small-Cell Lung Cancer
TNF-α	Tumor Necrosis Factor-alpha
IL-1β	Interleukin-1beta
PI3K	Phosphoinositide 3-kinase
CKD	Chronic Kidney Disease
PKD	Protein Kinase D
MAST	Microtubule-Associated Serine/Threonine Kinase
IDR	Intrinsically Disordered Region
